# Epidemiology of juvenile idiopathic arthritis in Oman

**DOI:** 10.1186/s12969-015-0030-z

**Published:** 2015-08-01

**Authors:** Reem Abdwani, Eiman Abdalla, Safiya Al Abrawi, Ibrahim Al-Zakwani

**Affiliations:** Department of Child Health, Sultan Qaboos University Hospital, P O Box 35, Al Khod, Muscat, PC 123 Sultanate of Oman; Department of Pediatrics, Royal Hospital, Muscat, Oman; Department of Pharmacology & Clinical Pharmacy, College of Medicine & Health Sciences, Sultan Qaboos University, Muscat, Oman; Gulf Health Research, Muscat, Oman

**Keywords:** Juvenile idiopathic arthritis, Children, Uveitis, Oman

## Abstract

**Background:**

There is a worldwide variation in the prevalence and subtype distribution of juvenile idiopathic arthritis (JIA) which may be affected by ethnicity and genetic factors. The purpose of this study is to determine the prevalence, subtype distribution and characteristic features of JIA among Omani children and to compare our results with other ethnic populations worldwide.

**Methods:**

A population-based, multicenter study among pediatric rheumatology clinics in the Sultanate over a 10 year period between 2004–2013. The diagnosis of JIA and identification of JIA subtypes was based on the ILAR 2004 revised criteria. The hospital charts of these patients were retrospectively reviewed and information was collected. All patients were screened for uveitis by an ophthalmologist using slit lamp examination during regular follow up visits.

**Results:**

The study included a total of 107 cases of JIA in Oman over the study period. Among the 107 patients, 71 % (*n* = 77) were girls with a female:male ratio of 2.5:1. The mean age of disease onset was 6.85 ± 3.86 years (range 1–13years) while the mean disease duration of 4.8 ± 2.9 years (range 1–11 years). The incidence of JIA was estimated at 2/100,000 with a prevalence of JIA of 20/100,000. The prevalence of JIA in girls was 28/100,000 while the prevalence in boys was 12/100,000. According to disease distribution, the most frequent subtype was polyarticular JIA rheumatoid factor negative (39.2 %) followed by oligoarthritis (31.8 %), systemic (17.8 %), polyarticular JIA rheumatoid factor positive (7.5 %). The unique feature of the Omani cohort is the lack of occurrence of uveitis.

**Conclusions:**

This is the first epidemiological JIA study conducted in Oman that highlights unique geographical disease phenotype. Compared to Western counties, there were higher frequency of polyarticular disease and lack of occurrence of uveitis. Further studies are needed to evaluate the implications of genetic, ethnic and environmental differences of disease expression.

## Background

Juvenile idiopathic arthritis (JIA) is an inflammatory disorder characterized by chronic arthritis. It is a clinical diagnosis in a child less than 16 years of age with arthritis that is defined as swelling or limitation of motion of the joint accompanied by heat, pain or tenderness for at least 6 weeks duration with other identifiable causes of arthritis excluded. JIA has been classified by the International League of Associations for Rheumatology (ILAR) into seven subtypes including systemic, oligoarticular, polyarticular rheumatoid factor (RF) positive and RF negative, enthesitis-related arthritis (ERA), psoriatic and “other” JIA [1]. The classification system was developed to identify clinically homogenous JIA subtypes to facilitate communication regarding epidemiology, therapeutics and outcomes globally [2].

JIA is the most common rheumatic illness in children in the Western world [3]. According to the literature, oligoarticular JIA (50–60 %), polyarticular JIA (30–35 %), systemic JIA (10–20 %) and ERA (1–7 %) are the most common JIA subtypes [2]. However, there is a worldwide discrepancy in the prevalence and subtypes distribution of JIA which may be affected by ethnicity and genetic factors [3]. There is paucity of data on epidemiology of JIA in children in Arabs. Therefore, the purpose of this study was to determine the prevalence, subtype distribution and characteristic features of JIA among Omani children and to compare our results with other ethnic population worldwide.

## Methods

We conducted a multicenter study among pediatric rheumatology clinics in the Sultanate Oman to identify patients with JIA over a 10-year period from 2004–2013. All the subjects included in the study were Omani children <13 years of age, being the cut of limit for the pediatric age group in most Arabian countries. Patients who had the signs and symptoms of other arthritis such as para-/post-infectious arthritis, connective tissue disorder, systemic vasculitis, malignancy, or metabolic diseases were excluded from the study after careful evaluation. The diagnosis of JIA and identification of subtypes of JIA was based on the ILAR 2004 revised criteria. Patients whose diagnosis was made prior to the use of the ILAR criteria were subsequently diagnosed as having JIA according to the ILAR criteria from a review of the medical records. All patients fulfilled the ILAR criteria for diagnosis of JIA at least six months prior to the inclusion into the study.

The medical records of the pediatric patients were retrospectively reviewed and information was collected including date of birth, age, gender, age at diagnosis, disease duration, joint involvement, JIA onset type and treatment. Immunological parameters collected included antinuclear antibodies (ANA), measured by indirect immunoflorescence and RF as detected by ELISA. Human leukocyte antigen (HLA) was evaluated on conventional lymphocytotoxicity assay in a few of the patients who had onset of arthritis after the age of 6 years. ANAs was considered positive if a titer of ≥1:80 was obtained on at least 1 clinic visit during the disease course. RF was considered to be positive when titers were >20 IU/ml. If only one test of RF was performed, as was the case in many patients, then the results of this test were used to assign a JIA subtype rather than apply the subtype category of “other JIA.” All patients were screened for uveitis by an ophthalmologist using slit lamp examination during regular follow up visits at 3, 6 or 12 monthly intervals as per the pediatric screening recommendation for uveitis in JIA [4].

The study was approved by Sultan Qaboos University, College of Medicine Ethics Comittee.

### Statistical analysis

Descriptive statistics were used to summarize the data. For categorical variables, frequencies and percentages were reported. Differences between countries were analyzed using Pearson’s *χ*^2^ test or Fisher’s exact test for cells <5. For continuous variables, mean and standard deviation were used to present the data. An *a priori* two-tailed level of significance was set 0.05. Statistical were performed using Stata, version 13.1 (STATA corporation, College Station, TX, USA).

## Results

Over the 10-year study period, 107 cases of JIA in Oman were identified. Among the 107 patients, 71 % (*n* = 77) were girls with a female to male ratio of 2.5:1. The mean age of disease onset of 6.85 ± 3.86 years (range 1-13years) with mean disease duration of 4.8 ± 2.9 years (range 111 years). The incidence of JIA was estimated at 2/100,000 with a prevalence of JIA of 20/100,000. The prevalence of JIA in girls was 28/100,000 while the prevalence in boys was 12/100,000.

According to disease distribution, the most frequent subtype was polyarticular JIA RF negative (39.2 %) followed by oligoarthritis JIA (31.8 %), systemic JIA (17.8 %), polyarticular JIA RF positive (7.5 %) and ERA (3 %) while psoriatic arthritis was detected in 0.9 % among our patient cohort. There were no patients who were classified in “others category”. The characteristic features of JIA subtypes, gender ratio, mean age at onset and ANA positivity is shown in Table 1. Oligoarticular JIA and systemic onset JIA had an earlier disease onset of 5 years than polyarticular JIA with disease onset at 8 years and ERA with mean age of onset at 12 years. With regards to sex distribution, polyarticular RF + JIA had the highest female to male ratio compared to both polyarticular JIA RF negative and oligoarticular JIA. While systemic onset JIA had equal sex distribution and patients with ERA were predominately males. Interestingly, patients with polyarticular RF + JIA had the highest frequency of positive ANA followed by oligoarticular and polyarticular RF – JIA. The highest inflammatory markers at disease onset was observed in patients with systemic onset JIA and polyarticular RF+ JIA.

**Table 1:** Characteristics of juvenile idiopathic arthritis (JIA) among children in Oman (*N* = 107)

JIA subtype	n (%)	Age at onset, years ± SD	F/M ratio	ANA+ (%)	Mean ESR, mm/h
Oligo JIA	34 (31.8 %)	5.3 ± 3.8	3.3/1	32.3	31.3
Poly RF –ve	42 (39.2 %)	8.0 ± 3.5	3.2/1	21.4	43.8
Poly RF + ve	8 (7.5 %)	8.8 ± 3.1	8/1	50	61.8
Systemic	19 (17.8 %)	5.0 ± 3.3	1/1	0	80.6
ERA	3 (2.8 %)	12.0 ± 2.6	0/3	0	62.6
Psoriatic	1 (0.9 %)	11	0/1	0	60
Others	0 (0 %)	na	na	na	na

*SD* standard deviation, *F/M* female/male, *ANA* anti-nuclear antibodies, *ESR* erythrocyte sedimentation rate, *Oligo* oligoarticular, *Poly RF* polyarticular rheumatoid factor, *ERA* enthesitis-related arthritis, *na* not available

The pattern of joint involvement in each subtype is shown in Table 2. Patients with oligoarticular JIA tended to have involvement of large joints of the lower limbs such as knees and ankles. Patients with ERA also had predominately lower limb involvement with sacroiliac joints, hip and knees affected in all cases. However the other JIA subtypes had involvement of both upper and lower limbs at variable frequencies.

**Table 2:** Joint involvement in juvenile idiopathic arthritis (JIA) subtypes in Omani children (*N* = 107)

Joint involved	Systemic (*n* = 19)	Oligo JIA (*n* = 34)	Poly RA + ve (*n* = 8)	Poly RA – ve (*n* = 42)	ERA (*n* = 3)
SI	0	0	0	3	1
Hip	2	0	0	4	3
Knee	10	29	8	39	3
Ankle	12	9	7	32	3
MTP	1	1	5	15	0
Spine	0	1	1	6	1
TMJ	0	0	1	2	0
Shoulder	1	1	0	1	0
Elbow	8	1	4	23	0
Wrist	7	3	8	33	0
MCP	1	0	4	20	0
PIP	2	2	4	15	0
DIP	0	0	0	4	0

*Oligo* oligoarticular, *Poly RF* polyarticular rheumatoid factor, *ERA* enthesitis-related arthritis, *SI* sacroiliac, *MTP* metatarsophalangeal joint, *TMJ* temperomandibular, *MCP* metacarpophalangeal joint, *PIP* proximal interphalangeal joint, *DIP* distal interphalangeal joint

The comparison of JIA subtypes in Oman with different ethnic populations is shown in Table 3. Systemic onset JIA was the most prevalent subtypes in Asian countries like Japan [5] and Arab origin like Saudi Arabia [6] while oligoarticular JIA subtype was most prevalent in western countries like Canada [7] and France [8]. However, polyarticular JIA, especially RF -, was most common in patients of Arab origin like Kuwait [9], Egypt [10] and Oman (current study) as well as in patients with African origin as in Zambia [11] and Nigeria [12].

**Table 3:** Distribution of juvenile idiopathic arthritis (JIA) subtypes among our cohort in comparison to other published literature

JIA subtype	Oman (*n* = 107)	Japan [5] (*n* = 1606)	France [7] (*n* = 67)	Canada [8] (*n* = 785)	Kuwait [9] (*n* = 108)	Egypt [10] (*n* = 132)	KSA [6] (*n* = 115)	Zambia [11] (*n* = 78)	Nigeria [12] (*n* = 78)	*P*-value
Systemic	17.8 %	54 %	8.9 %	13.6 %	29 %	13.6 %	44 %	14.1 %	7.7 %	<0.001
Oligo JIA	31.8 %	21 %	40.3 %	39.8 %	29 %	52.2 %	26 %	32 %	26 %	<0.001
Poly JIA	46.7 %	25 %		25.6 %	42 %	29.5 %	30 %	46.1 %	41 %	<0.001
Poly RF+	7.5 %	-	0	3.6 %	-	8.3 %	-	11.5 %	14.1 %	0.006
Poly RF-	39.2 %	-	22.4 %	22 %	-	21.2 %	-	34.6 %	26.9 %	0.001
Psoriatic arthritis	0.9 %	-	4.5 %	10.8 %	-	0	-	1.3 %	1.3 %	<0.001
ERA	2.8 %	-	17.9 %	8.8 %	-	4.5 %	-	6.4 %	23 %	0.002
Undifferentiated	-	-		1.3 %	-		-			0.019

*Oligo* oligoarticular, *Poly RF* polyarticular rheumatoid factor, *ERA* enthesitis-related arthritis

Percentages are column percents

Extra-articular feature of JIA were predominately seen in patients with systemic onset JIA which included fever (100 %), evanescent rash (66 %), generalized lymphadenopathy (50 %), hepatosplenomegaly (43 %) and serositis (34 %). A unique feature of our cohort is the absence of uveitis despite a 10-year follow up. Treatment modalities used for these patients included nonsteroidal anti-inflammatory drugs 97 % (*n* = 104), prednisolone 74 % (*n* = 80), methotrexate 61 % (*n* = 66) and biologic agents 34 % (*n* = 37).

## Discussion

Sultanate of Oman is an Arab country situated in the Middle East. It has a unique geographical variation; the coast is located by the Arabian Sea in the South and East and the Gulf of Oman on the Northeast. According to 2010 Omani census, the Omani population is estimated to be 1,957,336 of which 528,480 (27 %) are children below 14 years of age with equal M: F ratio. We report the result of a 10-year study, which is the first epidemiological study to be conducted on JIA in Oman.

In our cohort the incidence of JIA was estimated to be 2/100,000 with a prevalence of 20/100,000. Around the world, there are geographical differences in the epidemiology of JIA with incidence rates that varies from 1.6–23 and prevalence from 3.8–400/100,000 [13]. The variation in results around the world may be associated with many reasons. Since classifications have been proposed and changed over the last decades, differences in data found with use of certain classifications may reflect changes due to time rather than real differences. Time may also have affected the methodological quality of the studies and how results are presented. Geographical differences may also be linked with the type of study which could be clinic-based study or population-based study and the case ascertainment methods used, which have varied between accurate systematic health visits attended and survey-based methods on questionnaires to health practitioners. Therefore, determining if the differences reported in the prevalence and incidence are true differences is difficult because of the heterogeneity of classification used, time, geographical zone, and methodology used [3].

Ethnic differences in the prevalence of JIA have also been observed in various populations. As examples, the prevalence of JIA is less in blacks, Asians, and Indian ethnicity than in Caucasian children in North America [14] and is higher in the native populations than among Caucasians among Canadian children [15]. Furthermore, in a multiethnic cohort from Toronto, Canada, those from European descent were associated with a significantly increased risk of developing JIA [8]. In addition to ethnic diversity in epidemiology of disease, there is a significant differences in JIA subtype distribution around the world. Our study included patients who were Omanis with no other ethnic background. In our cohort, there was a relative paucity of oligoarticular disease with a higher prevalence of polyarticular JIA compared to the Western countries. The relative rarity of oligoarticular JIA in non-European populations has also been documented in JIA patients from Kuwait [9], Turkey [16], Thailand [17], Japan [5] and South Africa [18] as well as in African American children in the USA [14].

Oligoarthritis has a striking age at onset distribution with a peak incidence between 2 and 4 years with a small proportion of children having the disease onset after this time [19].

However, in our cohort of patients, the mean age of disease onset was older at 5.4 ± 3.8 years. In Western countries, the sex ratio of 5–6:1 is observed in patients with oligoarticular disease with uveitis, while in our cohort the sex ratio was 3.3:1 [19]. Therefore, our cohort has an older age of onset onset and relative paucity of girls than the Western population. The pattern of joint involvement in our patients with oligoarticular JIA is comparable to those reported in the literature as it predominately affects large joints of the knees (85 %) and ankles (26 %) [20].

Patients with extended oligarticular JIA had involvement of wrists (8.8 %) and elbows (3 %).

Polyarticular JIA typically has a biphasic age of onset with an early peak between the ages of 1 and 4 years, and a later peak at the ages of 6 and 12 years [19]. In our cohort of polyarticular JIA (*n* = 50), there was predominately a late onset peak in the majority of the children (62 %), while a minority (14 %) had an earlier onset peak of disease onset. Polyarticular JIA is predominately a disease of girls, the sex ratio of our cohort matches that reported in the literature 4:1 [19]. The pattern of joint involvement in polyarticular RF – showed that there was a greater involvement of lower limbs than upper limbs with knees and ankles affected at 92 and 88 % of the cases while wrists, elbows and small joints were affected in 78, 55 and 35 % of cases, respectively. Patients with polyarticular RF+JIA, had equal involvement of both lower and upper limbs with knees and ankles involved in 100 and 88 %, respectively, while wrists and elbows were involved in 100 and 50 % cases, respectively.

Systemic onset JIA characteristic features in our cohort matched those reported in the literature in terms of frequency, equal sex distribution and mean onset age of onset [19]. Any number of joints can be affected at onset of disease. The pattern of joint involvement in our patients with systemic onset JIA included involvement of both upper and lower limbs in a symmetrical distribution with knees and ankles involved in 52 and 63 % of cases while elbows and wrists were involved in 42 and 37 % of cases, respectively. Similarly, our cohort of patients with ERA matched the pattern found worldwide with predominately a male disease affecting the lower limbs with relative late childhood onset.

JIA associated uveitis occurs in 10–20 % of all cases [19]. Typically, uveitis occurs most commonly in young girls with oligoarticular onset JIA, with ANA positivity (78 to 90 %) while it occurs in 7–15 % of polyarticular and rarely in systemic onset variety. The greatest risk for developing uveitis occurs within the first 2 years after the onset of JIA, and the risk declines by 7 years after the onset of arthritis. The unique feature noted in our cohort of JIA population was the absence of uveitis including the oligoarticular JIA subtype characterized with the following risk features for developing uveitis including mean onset of disease at 5.2 years with high female to male ratio of 3.3:1 and ANA positivity in 32 % of cases. Perhaps the older age of onset, relative paucity of girls and relative lack of frequency of positive ANA can explain the uncommon occurrence of uveitis as a complication in our cohort. Geographical variations in the incidence of uveitis in JIA have been reported around the world [21–23]. In a series of 89 JIA patients from India, there were no cases of uveitis in patients with oligoarticular disease with ANA positivity [21]. In a study in New Zealand, only one of 55 JIA children developed uveitis [22].

Atypical clinical features of uveitis were described in a study from Costa Rica with four of the 48 patients with JIA developing uveitis, and all of them were anti-nuclear antibodies (ANA) negative and >7 years of age at arthritis onset [23].

This study is not without limitations including its retrospective nature. Furthermore, this study was also not fully generalizable as it was hospital- and not-population based. The patients’ case-mix included all patients that were referred to a pediatric rheumatology clinic. However, it may not have included the “milder cases” that have not been correctly diagnosed and referred by a general practitioner and paediatrician. Perhaps, only the more “obvious” polyarticular type of disease were diagnosed correctly and referred to a tertiary hospital. This could have resulted in underestimation of our parameters due to referral bias. Despite this, a previous population based cohort study compared with those from the referral-based cohort, found no significant differences with regard to disease duration, frequency of uveitis, subgroup distribution, disease activity, and number of tender/swollen joints [24]. The other limitation is recruitment of children with JIA who were below 13 years of age, and did not include patients up to 16 years which is the defined as JIA according to ILAR classification because of the government policy on the cut off age limit in the pediatric population in the hospitals in Oman; therefore, there was underestimation of total number of patients.

## Conclusion

This is the first epidemiological JIA study conducted in Oman that highlights unique geographical disease phenotype. We conclude that the characteristics of JIA in Omani children with are different from those of European and North American origin with higher frequency of polyarticular disease and lack of occurrence of uveitis. Taken together, we suggest that the differences in immunogenetic background of ethnic groups may account for the differences in expression of disease. There is a need for further studies from different ethnic groups and geographic locations in order to improve our knowledge about how genetic and environmental differences affect JIA expression.

## Abbreviations

JIA: Juvenile idiopathic arthritis
ILAR: International League of Associations for Rheumatology
RF: Rheumatoid factor
